# Interlayer Defect Detection in Intra-Ply Hybrid Composite Material (GF/CF) Using a Capacitance-Based Sensor

**DOI:** 10.3390/s22082966

**Published:** 2022-04-13

**Authors:** Khalid Alblalaihid, Saleh A. Alghamdi, Anas Alburayt, Saif H. Almutairi, Ahmed Alwahid, Meshal Abuobaid, Sabri Alkhibari, Khaled S. Almutairi, Ibrahim M. Alarifi

**Affiliations:** 1 Space and Aeronautics Research Institute, King Abdulaziz City for Science and Technology (KACST), Riyadh 11442, Saudi Arabia; alblehed@kacst.edu.sa (K.A.); abareet@kacst.edu.sa (A.A.); shmutairi@kacst.edu.sa (S.H.A.); aweheed@kacst.edu.sa (A.A.); mabuobaid@kacst.edu.sa (M.A.); salkhaibari@kacst.edu.sa (S.A.); kmutiri@kacst.edu.sa (K.S.A.); 2 Department of Mechanical and Industrial Engineering, College of Engineering, Majmaah University, Al-Majmaah, Riyadh 11952, Saudi Arabia; i.alarifi@mu.edu.sa

**Keywords:** sensing, matrix cracking, intra-ply hybrid composites, glass fibers, carbon fibers

## Abstract

Combining two types of reinforcement fiber in a common matrix may lead to different failure modes such as micro-cracks between the layers when the structure is subjected to lower stress levels. Real-time damage detection should be integrated into the hybrid composite structure to provide structural integrity and mitigate this problem. This paper outlines the working mechanisms and the fabrication of an integrated capacitive sensor in an intra-ply hybrid composite (2 × 2 twill weave). Uniaxial tensile and flexural tests were conducted to characterize the proposed sensor and provide self-sensing functionality (smart structure). The sensitivity and repeatability of the capacitive sensor were measured to be around 1.3 and 185 µΔ*C*/*Co*, respectively. The results illustrate that onset of damage between layers can be detected by in situ monitoring. It can be seen that the initial damage was detected at the turning point where the relative change in capacitance begins to reduce while the load increases. Finite element modeling was also constructed to analyze the test results and explain the reasons behind the turning point. It was shown that the carbon yarns experienced high transverse shear stress (τ_xz_) in the crimp region, leading to inter-fiber cracks.

## 1. Introduction

There is an increasing demand for fiber-reinforced polymer composites (FRPC) in the sporting goods, automotive, and aerospace industries. FRPC has several advantages such as high modulus and strength to weight ratio and suitable temperature and chemical resistance. Glass fiber is the most common material used as reinforcement and accounts for more than ninety percent of the fiber reinforced polymer composite (FRPC) market [1]. In addition, it has good flexibility and low cost. In recent years, extensive research has been conducted to optimize the weight and improve the glass fibers’ mechanical properties by hybridizing the glass with carbon fibers [2,3]. The improvement is due to the carbon fibers having higher stiffness and lower density than glass fibers [4]. Investigations into the main effect have been conducted on the impact of structural characteristics (such as the number of plies, their orientation, and the sequence of hybrid stacking) [5]. It is necessary to select suitable fiber types for hybrid composites in order to obtain the highest potential mechanical strength, either interwoven (intra-ply) or layered (inter-ply) [6]. Various hybrid composites of interlayer and intralayer warp-layers were investigated with carbon and glass fibers for the impact of layer placing on the behavior of composite laminates including interlayer and intralayer warp layers with carbon and glass fibers [7,8,9]. A previous study discovered that inter-ply and intra-ply deformation both contributed to increased fracture toughness of the prepreg laminate [10,11,12]. However, FRPC is an anisotropic material and has a low interlaminar strength, which is generally subject to degradation of the overall mechanical properties due to micro-cracks and delamination between layers. Additionally, hybrid carbon and glass fiber composites have two types of elastic moduli, which can cause different failure modes such as delamination between layers and fragmentation [13]. Therefore, implementing a real-time damage sensing technique to monitor changes in the geometric properties of hybrid composite structures would significantly preserve the structural integrity over time.

The importance of electrical conductivity in the aerostructure is that the damage produced by lightning strikes can be minimized and an embedded sensor (e.g., piezo-resistive sensor) can also be used to detect damage. Rajesh et al. described a lightning protection system (LSP) based on a conductive covering and, as part of their investigation, two distinct conductive layers (hybrid and metallic) were placed on a carbon fiber reinforced polymer (CFRP) panel in order to test the materials’ damage resistance under the conditions of simulated LSP [14]. All techniques, except the conductive coating strategy, were used to increase the conductivity of the CFRP composites across their whole thickness. The conductive tufting/yarn method, which is unique, increased the material’s through-thickness conductivity and mechanical qualities [15].

Carbon nanotubes (CNTs) dispersed in an epoxy matrix, in conjunction with carbon fibers in intra-ply hybrid laminates, have been used to create a three-dimensional electrical sensory network in the composites [16]. It has been discovered that the ply lay-up sequence and laminate thickness significantly impact both the mechanical performance and the damage processes of hybrid composites [17]. With electro-flocking technology, innovative electrically conductive glass/carbon/inter-ply/intra-ply fiber hybrid reinforced epoxy composite laminates have been investigated for piezo-resistive damage sensing applications [18]. When the matrix material of a carbon fiber composite is conductive, the reliability of the sensor is increased [19]. Interlaminar shear deformation had a role in altering the previously installed electrical network [20]. By interleaving the carbon nanotube film prepreg between layers using an insulating epoxy adhesive, it is possible to create in situ strain sensors in cross-ply carbon epoxy composites [21]. However, it has been reported that there are several challenges to using the carbon nanotubes technique such as an electrical tunneling effect and accumulating resistance associated with small cracks [22]. Even though the nanomaterial that is integrated with the matrix has good sensitivity, the main load in the composite is carried by fiber reinforcement, which can lead to the provision of limited information [22]. 

Employing optical fiber is another technique that can be used as a real-time sensor where light is produced through the emitter as it travels along a fiber and reflected light will be received to detect changes in strain. Oromiehie et al. reported that the localized and multiplexed sensing capabilities of fiber Bragg grating (FBG) sensors make them particularly well suited to be integrated into composite materials [23]. However, the process of integration of the optical fiber sensor into the FRPC requires perfect adhesion to monitor strain within the structure. Additionally, it has been reported that the fiber optic sensor embedded into the composite is susceptible to damage induced by bending [24,25]. 

The number of carbon fiber plies used in the interlaminar hybrid composites influences the mechanical and electrical characteristics of the composites. As a result, the general aim of this study is to report on the damage sensing capabilities of hybrid intra-ply carbon/glass laminated composites under loading situations and to provide recommendations. The novelty of the work presented in the paper is the initial fabrication and basic characterization of integrating a capacitive sensor into hybrid composites using yarns of carbon fiber as electrodes and glass fibers as a dielectric to provide self-sensing functionality (smart structure) with a linear strain–capacitance relationship. The study used an intra-ply hybrid composite to hybridize carbon fibers into glass fibers where the carbon yarn was used in the same ply as the glass fibers.

In the present work, the results for a performance evaluation are presented for this new form of an embedded capacitive sensor for intra-ply hybrid carbon/glass (CF/GF). The issue associated with misalignment imperfections during the lay-up process of the intra-ply hybrid composite is first presented and followed by a demonstration of the hybridization methods. The method used to complete a mechanical and electrical characterization of the embedded sensor is then presented. Based on the characterization results, a number of key findings were evaluated including onset damage, sensitivity, and repeatability. Finally, stress analysis was conducted using commercial finite element software (COMSOL) to clarify and explain the experimental results.

## 2. Materials and Methods

**Figure 1:**
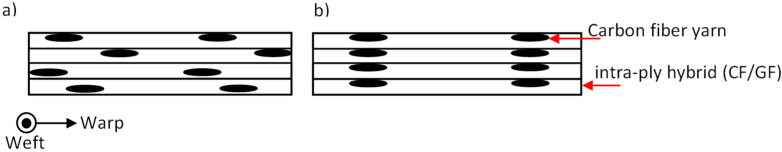
Schematic diagram of the cross-section of the intra-ply hybrid composite. (**a**) Carbon yarns were incorrectly aligned on top of each other due to misalignment of the plies, (**b**) the carbon yarns were integrated with glass fiber aligned perfectly on top of each other.

The central aim of this project was to develop and study the intralayer defect detection in intra-ply hybrid carbon/glass (CF/GF) fiber composite material. To complete this study, several assumptions were made. All hybrid composites manufactured in the study consisted of seven plies stacked with the same orientation (weft direction). The carbon yarn was integrated into three plies of the hybrid composite. In reality, it is expected that there will always be misalignment imperfections during the lay-up process where the carbon fiber yarns that are integrated with glass fiber to form the intra-ply hybrid composite will not align perfectly on top of each other, as described in Figure 1a. Therefore, to simulate worst-case scenarios of interlayer defect detection of hybrid composites as a result of using two different types of reinforcement, the plies integrated into the carbon fiber were separated with unmodified glass fiber ply to transfer efficient stress from the matrix and glass fiber to the carbon yarn. The tensile test was conducted by applying load along the direction of carbon fiber filament yarns. 

The working principle of the capacitive sensor that detects a defect in the hybrid composite is presented in Section 2.1. To provide and validate the concept of the proposed sensor, the fabrication process of the intra-ply hybrid fabric and the capacitive sensor is described in Section 2.2.

### 2.1. Working Principle of the Proposed Sensor

**Figure 2:**
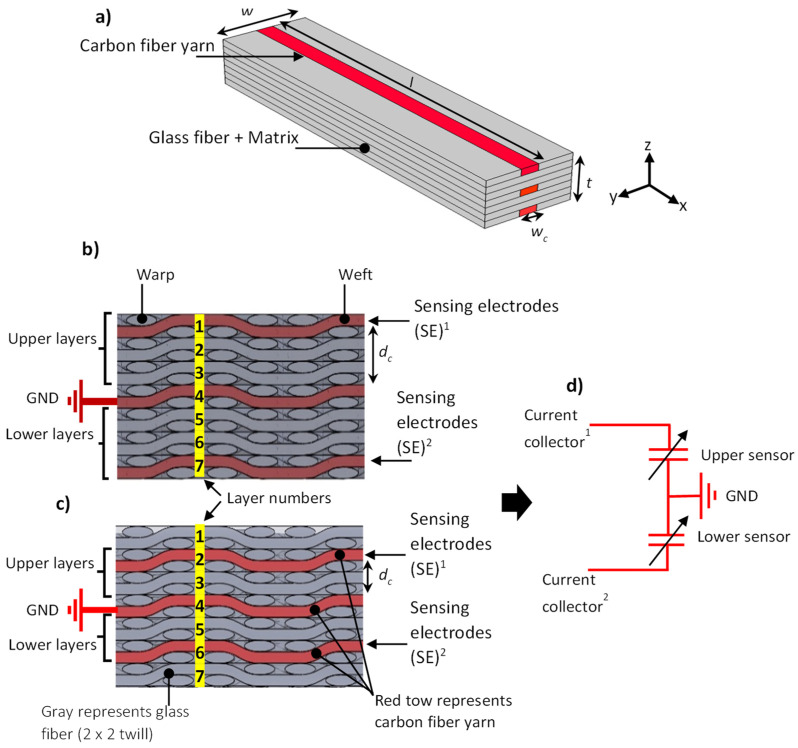
(**a**) Layout of self-sensing intra-ply hybrid composites, (**b**,**c**) show the sensor configurations for type-A and type-B, (**d**) electrical circuit of the embedded sensor. (SE)^1^ and (SE)^2^ represent upper layer and lower layer sensing electrodes respectively.

The new self-sensing intra-ply hybrid composite was based on parallel plate capacitance. Two differential capacitive sensors consisting of a three-electrode system were embedded in the laminated structure, as shown in Figure 2a. To form the capacitance in the intra-ply hybrid composites, the yarns of carbon fiber were used as electrodes, and insulated and separated by glass fiber, which worked as a dielectric. The capacitance is given by Equation (1):

(1) $$C_{o}=\varepsilon_{r}\varepsilon_{o}\frac{w_{c}l}{d_{c}}\;$$

where *C_o_* is the initial capacitance; *ε_r_* is the effective permittivity for glass fiber reinforced epoxy composite; *ε_o_* is the vacuum permittivity; *d_c_* is the thickness of the dielectric; *w_c_* and *l* are the width and length of electrodes, respectively. All parameters are defined in Figure 2. 

When a hybrid composite material is subjected to applied tensile stress, the embedded sensor will deform in all directions (*d_c_*, *w_c_* and *l*), which in turn leads to a change in the capacitance value, as specified in Equation (1). Then, the changes in electrical capacitance associated with applied stress can be measured in real-time. 

To perform a sensitivity analysis of the sensor and study the onset of tensile failure for a low elongation fiber (carbon fiber) in a hybrid composite, two different sensor configurations, which had different stacking sequences of the intra-ply hybrid composite, were considered, as shown in Figure 2b,c. The electrodes (the carbon fiber yarns) were separated by two layers of glass fiber that were integrated into layer numbers 1, 4, and 7, as defined in Figure 2b. To form a dual differential capacitive sensor, the middle electrode (layer 4) was connected to ground (GND) and upper (layer 1), and lower (layer 7) electrodes were connected to the current collector. Figure 2c illustrates the second configuration where the electrodes were separated by one layer of glass fiber and integrated into layer numbers 2, 4, and 6. The equivalent electrical circuit of the differential sensor for both configurations is illustrated in Figure 2d. To evaluate the strain sensitivity of the proposed sensor, the gauge factor was estimated experimentally based on Equation (2).

(2) $$k=\frac{\Delta C/C_{O}}{{ɛ}_{x}}$$

where *k* is the gauge factor; Δ*C*/*C_O_* is the relative change in capacitance; and *ɛ_x_* is the longitudinal strain along the carbon fiber yarn.

### 2.2. Sensor Fabrication

**Figure 3:**
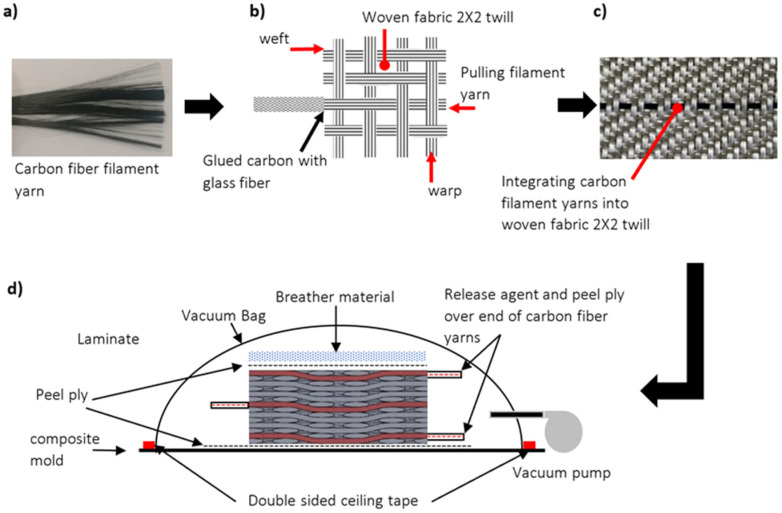
Fabrication process of the intra-ply hybrid fabric and capacitive sensor: (**a**) an image of continuous carbon fiber yarn, (**b**) schema showing the process of integrating continuous carbon fiber yarn into fiberglass woven fabric 2 × 2 twill 280 g/m^2^, (**c**) an image of the intra-ply hybrid fabric produced, and (**d**) schematic presentation of vacuum bagging system.

In order to produce intra-ply hybrid composites, continuous carbon fiber tow with 3k filaments was used (Torayca^®^ T300, 3k, Toray Composite Materials America, Inc. (CMA), Tacoma, WA, USA), as can be seen in Figure 3a. According to the technical data sheet, the carbon fiber filament used in this study has a high tensile strength of around 3750 MPa [26] and also has good electrical conductivity, which enabled its use in this work as an electrode material (see Table 1). The carbon fiber yarns were incorporated into the glass fiber (2 × 2 twill, FK 144, Interglas-92125, Haufler Composites, Blaubeuren, Germany), which works as a dielectric material between electrodes. The electrical and mechanical properties of carbon fiber filament and 2 × 2 twill fiberglass composite are presented in Table 1. The hybridization was achieved through two steps. First, the tip of a carbon yarn was glued to one of the glass fiber yarns in the weft direction using spray adhesive (3M hi-strength 90, USA), as shown in Figure 3b. Then, the glass fiber yarn was pulled from the other end to replace the glass yarn with carbon and have the same weave pattern, as shown in Figure 3c. 

In this study, the sample was manufactured using a hand lay-up process, which can be summarized in several steps, as shown in Figure 3d. First, a peel ply (85 g/m^2^, China Pioneer New Material Group Ltd., Jiaxing, China) was placed on the mold, which works as release fabric and prevents the laminate from sticking to the mold. On top of the peel ply, the seven layers were stacked and impregnated with L285 epoxy resin produced by HEXION Specialty Chemicals Inc., Stuttgart GmbH (at a laboratory temperature of 25 °C). The weight ratio of epoxy resin/hardener matrix material was 100/40. H287 hardener manufactured by HEXION was used where the gel time lasted longer, which helped to simplify the preparation of the intra-ply hybrid composite samples during the lay-up process.

The glass fiber becomes translucent when impregnated and fully wetted with the epoxy, making the carbon yarns visible between layers. This advantage allows the three carbon fiber yarns that are integrated in the layers to be aligned on top of each other. Release agent (Formula five^®^ mold release wax, REXCO, Conyers, GA, USA) was applied at the end of carbon fiber yarns that were to be electrically connected and wrapped with peel ply to prevent sticking in the mold. A breather material provided by Tygavac Ltd. was used and separated from the laminate using peel ply to absorb excess epoxy. The pressure was applied to the laminate using a vacuum bagging process where the vacuum pressure was around 800 mbar, and cured at ambient temperature for one day. 

Then, the specimens were cut according to ASTM D3039 using an oscillating cutting tool to produce test pieces for tensile tests and to characterize the electromechanical properties, as described in Section 4.1 and Section 4.2.

## 3. Experimental Setup

Two types of experiment were designed to investigate the performance of the self-sensing intra-ply hybrid composites. First, the behavior of the embedded sensor system (upper and lower sensor) when subjected to direct tensile stress was investigated. This initial investigation determined the sensitivity of the embedded sensors and the onset of tensile failure strain for a low elongation fiber (carbon fiber) in a hybrid composite for two sensor configurations (described in Section 2.1). Second, the embedded sensor was investigated by using a cyclic flexural test (three-point bending test). In this investigation, the repeatability of strain sensing for the embedded senor was investigated, and early onset of tensile failure stress for a low elongation fiber (carbon fiber) was measured, which will be described in detail (see Section 4.2).

### 3.1. Experimental Setup for Tensile Tests

**Figure 4:**
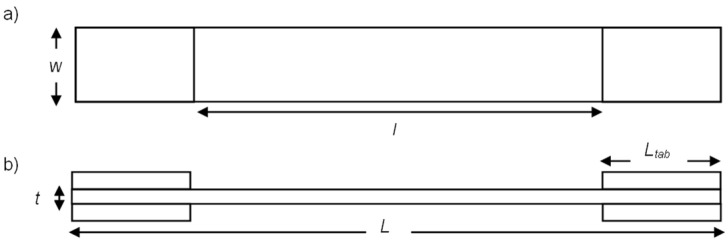
Main geometric parameters of the specimen according to ASTM D3039: (**a**) top view and (**b**) front view.

In order to initially characterize the sensing capability of two different sensor configurations (type A and B), the samples were subjected to direct tensile stress. The 40 mm long glass/epoxy tabs were bonded to the ASTM D3039 specimens using L285 epoxy resin to protect the sample from damage by the grips and provide electrical protection of the sensing electrodes. The geometric parameters that define each of the structures of tensile test specimens are presented in Figure 4 and detailed in Table 2.

**Figure 5:**
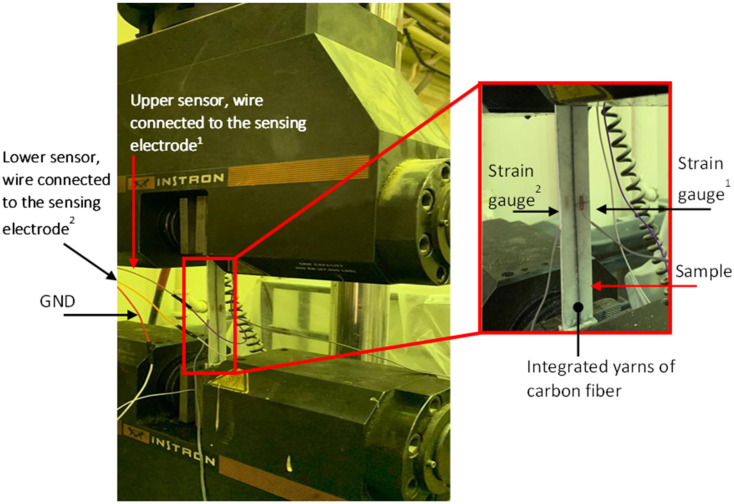
An image of the experimental setup used for the mechanical test on an Instron test machine (67500LBS). They represent the order number e.g., Strain guage^1^ and Strain guage^2^, also Upper sensor electrode^1^ and Lower electrode^2^.

To accurately measure the onset of tensile failure and the strain and stress for a low elongation fiber (carbon fiber) in the intra-ply hybrid composites, the capacitance and the force and strain were recorded during the tensile test. The axial force acquired from the loadcell was converted to axial stress. The specimens were equipped with two electrical strain gauges that were adhered to the upper and lower surfaces to measure the strain. The strain gauge transducers (Measuring Instruments Laboratory Co., Tokyo, Japan, FLA-5-11-1L) were located at mid gauge length, as shown in Figure 5. The axial force and the strain data were acquired from a universal testing machine (300 kN load cell, Instron, Norwood, MA, USA) with a sampling rate of 1 Hz. The speed of the crosshead was set at 1 mm/min. The electrical connection for the embedded capacitive sensor was made by twisting a carbon fiber yarn around the wires. Then, the wires were connected to a commercially available integrated circuit (PCap04, ams AG) to measure the relative change in capacitance with a sampling rate of 2.5 Hz.

### 3.2. Experimental Setup for Cyclic Flexure Tests

**Figure 6:**
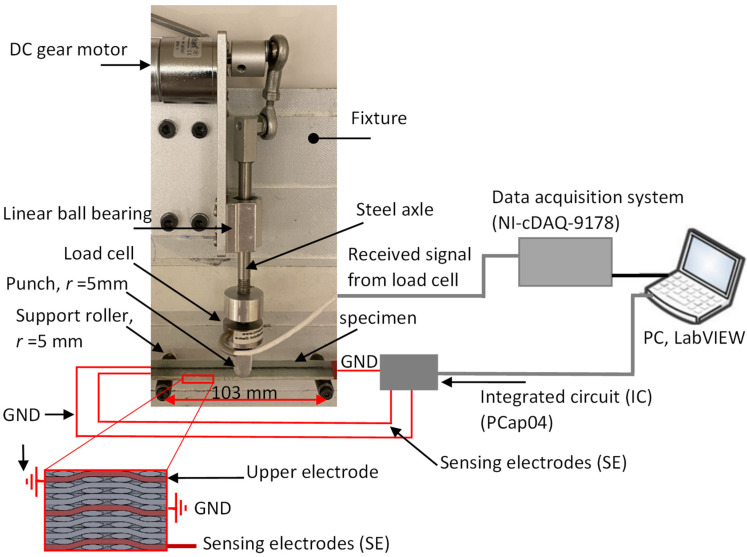
Configuration and experimental setup of the cyclic three-point bending test.

To complete the cyclic 3-point bending test experiment, test equipment developed at KACST was utilized, as shown in Figure 6. This equipment provides harmonic motion at up to 5 rpm using a geared DC motor and has an adjustable crosshead where the maximum displacement amplitude is 10 mm. The applied force was measured using a universal load cell (LCM202-500N) from OMEGA, integrated into the steel axle, as shown in Figure 6. The signal from the load cell was acquired by National Instruments hardware (NI-cDAQ-9178) and LabVIEW. Then, this force was used to determine the flexural stress (*σ_f_*) using Equation (3) [24]. The deflection at the center of the beam was approximated using Young’s modulus (*E_x_* ≈ 18.7 GPa), which was measured experimentally in tensile mode (Section 4.1), see Equation (4) [30].

(3) $$\sigma_{f}=\frac{3PL}{2wt^{2}}$$

(4) $$\delta =\frac{PL^{3}}{4wt^{3}E_{x}}$$

A small punch with a diameter of 10 mm was attached to the load cell to introduce a force between the two support rollers. The specimen was mounted on the top of 10 mm diameter support rollers with a span of 103 mm. The capacitive measurement was carried out using a commercially available integrated circuit (PCap04) from ams AG with a sampling rate of 5.8 Hz.

## 4. Performance Evaluation

### 4.1. Performance in the Longitudinal Direction

**Figure 7:**
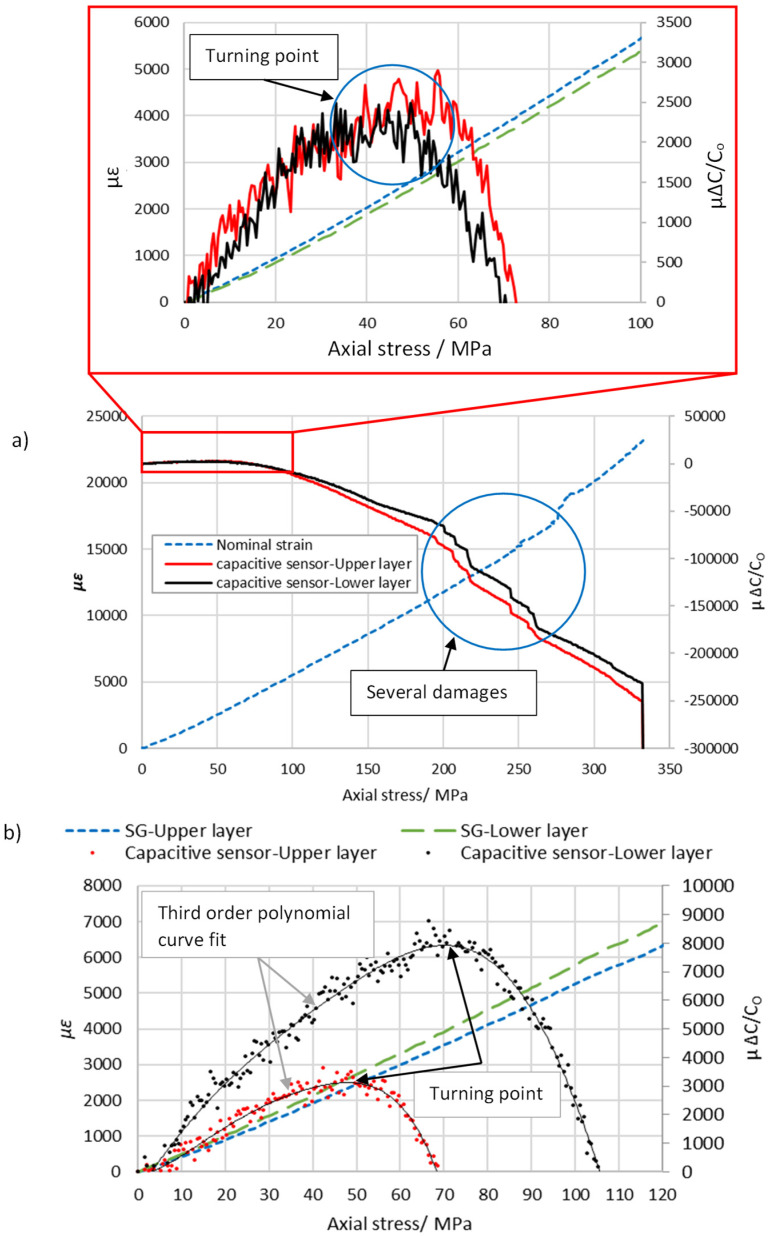
Graph of relative change in capacitance and nominal strain against the axial stress for the type A configuration, (**a**) sample A−1 pulled to breaking point; and the inset in graph (**a**) shows an enlarged view where the ranges of the *x*- and *y*-axes were limited to 100 MPa and 6000 µɛ. (**b**) Experimental result for sample A−2 where ranges of the *x-* and *y*-axes were limited to 120 MPa and 8000 µɛ.

In order to perform the uniaxial tensile tests for the two sensor configurations described in Section 2.1, type-A and type-B, the experimental setup described in Section 3.1 was utilized. The sample (A-1) was loaded onto the tensile test machine to obtain the relative change in capacitance and stress–strain curves. The sample was pulled to breaking point and the results obtained are illustrated in Figure 7. An initial assessment of Figure 7a showed that the relative change in capacitance remained constant at the beginning and then started to reduce up to failure. However, a portion of Figure 7a was enlarged where the ranges of the *x-* and *y*-axes were limited to 100 MPa and 6000 µɛ, as can be seen in the inset to Figure 7a. It was observed that the relative change in capacitance increased up to the turning point and, after that, began to decrease with increasing axial stress. It is obvious that the capacitive sensors detected the consecutive damage of fibers during tensile tests up to failure. Therefore, this research will focus on detecting the onset of damage of low elongation fiber for intra-ply hybrid composites. 

To ensure reproducibility, the tensile test was repeated with a new sample (A-2) identical to the sample (A-1) but manufactured at a different time. Figure 7b shows the tensile test results for sample A-2. On inspection, it can be seen that the shape of the curves for the capacitive sensors of the upper and lower layer were similar to those collected experimentally for sample A-1. To find the axial stress at the turning point for both tests, a third-order polynomial regression was implemented to fit the experimental test data, and then the root of the first derivative was determined (see Equation (5)).

(5) $$\frac{\partial ∆C/C_{O}}{\partial \sigma_{a}}=0$$

where ∆*C*/*C_O_* and *σ_a_* are the relative change in capacitance and axial tensile stress, respectively.

**Figure 8:**
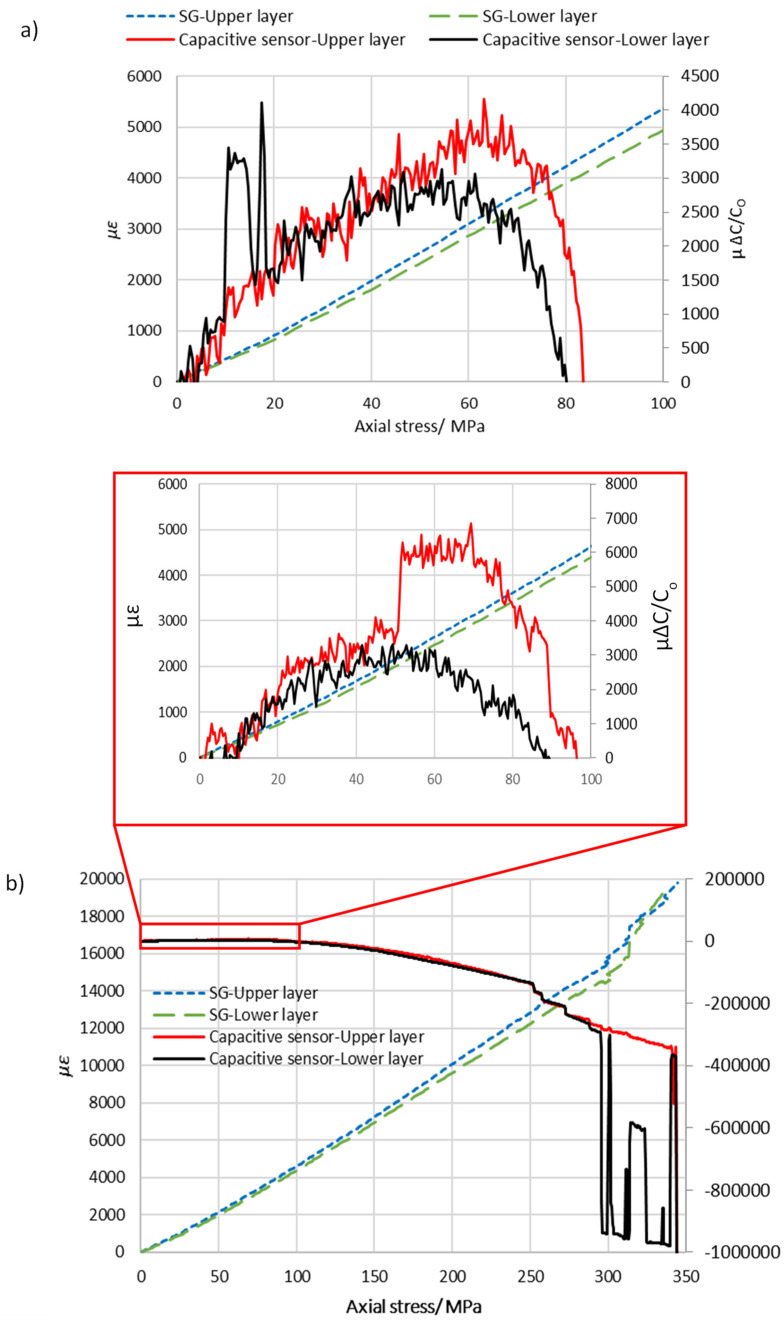
A plot of relative change in capacitance and nominal strain against the axial stress for the type-B configuration. Graph (**a**) shows the experimental results for sample B-1, where ranges of the x- and y-axes were limited to 100 MPa and 6000 µɛ. (**b**) The result shows sample B-2 pulled to breaking point and the inset in graph (**b**) shows an enlarged view where the ranges of the x- and y-axes were limited to 100 MPa and 6000 µɛ.

Two samples using the second sensor configuration (type-B) were tested, and the results are plotted in Figure 8. Changing ply numbers between electrodes as in type-A and -B led to changes in the dielectric thickness, which in turn changes the measurement of initial capacitance based on Equation (1). The averages of the initial capacitance for type-A and type-B were measured to be 53.8 pF and 73.3 pF, as shown in Table 3. The capacitive sensors exhibited linear sensitivity to the axial stress (σ_a_) up to the turning point. Therefore, the gauge factor (k) was experimentally calculated over a strain range of 2500 µɛ using Equation (2). It was found that there was not a big difference between the estimated gauge factor for type-A and -B, which was around 1.3. This gauge factor is relatively high compared with other structural health monitoring systems based on linear capacitive sensors such as 0.92 [31]. 

A slight difference was observed between the results obtained from samples A-1 and A-2 such as the difference in elastic modulus, which was around 7.4%. This observed difference may be attributed to the higher thickness of sample A-1, resulting from inefficient vacuum pressure on the vacuum bagging process [32]. Residual stresses can also occur in composite structures as a result of the curing process, which can lead to an alteration in the mechanical properties, specifically for thick plies [33]. Furthermore, the results show that the type-A specimen configuration had a lower strain and axial stress than type-B at the turning point. The axial stresses of type-A and B at the turning point were 49.7 MPa and 57 MPa, respectively. This indicates that the laminate stacking sequence of the intra-ply hybrid composite affected the stress value at the turning point. The turning point characteristics and gauge factor are summarized in Table 3. 

### 4.2. Cyclic Flexural Test

It is important for a multifunctional material that the self-sensing has a stable and repeatable measurement before and after the structure is exposed to deformation. Therefore, the repeatability of the strain sensing was conducted through loading and unloading the structure by harmonic excitations. In addition, to further investigate interlaminar damage detection for intra-ply hybrid composites that are subjected to bending, the three-point bending test was performed. 

**Figure 9:**
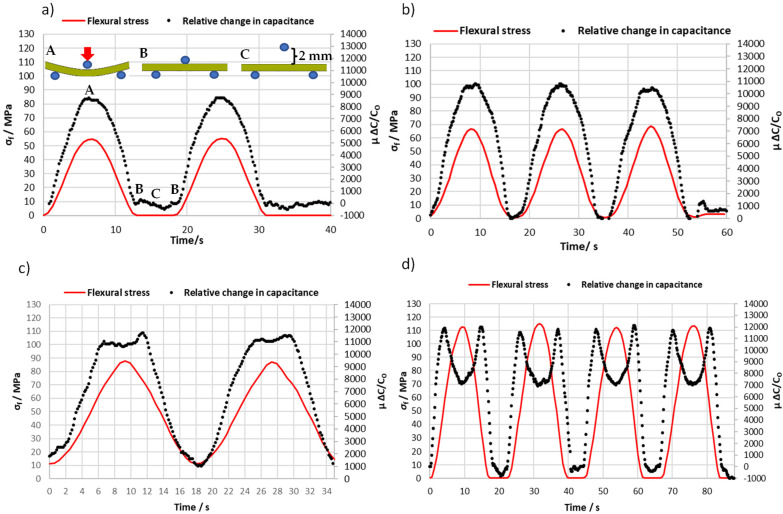
Time history of flexural stress and relative change in capacitance under variable amplitude loading: (**a**) 53.5 MPa where A, B and C describe the status of sample during the cyclic flexural test (refer to the experimental set-up in Figure 6), (**b**) 65.4 MPa; (**c**) the sample was initially loaded of 11 MPa up to 86 MPa, and (**d**) the sample was loaded four times at 112.7 MPa.

The flexural test was only conducted on the type-A intra-ply hybrid composite configuration. This is because the carbon yarn was integrated into the top and bottom surfaces of the composite layer, which is subjected to maximum compression and tensile stress during the flexural test. The relative change in capacitance of the lower sensor was only recorded to obtain the effect of the tensile stress side of the flexural test. The upper electrode was grounded to shield and minimize the external interference from the metallic crosshead when the punch approached the surface of the specimen, as shown in Figure 6. Figure 9 illustrates a series of cyclic flexural tests performed on one sample with a width of 22.22 mm and thickness of 1.95 mm. The maximum cyclic stresses applied to the specimen did not exceed 30% of the ultimate static bending stress (460 MPa) [28], as shown in Table 4. To examine the interference between the sensor and metallic crosshead after being grounded to the upper electrode, the punch was distanced by around 2 mm from the sample’s surface, as can be seen in the result in Figure 9a. After that, the sample was loaded two times at 55 MPa. It is clear that the interference was reduced when the small punch approached and was pulled away from the surface. Figure 9b shows the sample initially loaded with a small force up to 66.6 MPa and repeated three times. It was observed that the turning point started to appear at 63.7 MPa. To further investigate the turning point, the load was increased to 87.9 MPa where 11.2 MPa initially loaded the sample, as shown in Figure 9c. Finally, the load was cycled four times at 112.7 MPa and it was shown that the relative change in capacitance sensor dropped by around 5000 (µ Δ*C*/*Co*) over the turning point. Controlling crosshead displacement (*δ*) of the flexural test machine needs to be adjusted where the same sample needs to be loaded into the machine each time. This could cause a variation in the flexural stress of the turning point when the sample is loaded at 65.4, 86, and 112.7 MPa. However, the sample was loaded four times, as shown in Figure 9d, and the turning point appeared when the load was applied and removed in the same manner. The repeatability was estimated with eight measurements of turning points and the results indicate that the integrated capacitive sensor had good repeatability, which was around (11,837.5 ± 185) µΔ*C*/*C_O_*. Further details of the results are given in Table 4, which presents the electrical and mechanical results obtained from the three-point bending test under variable amplitude loading.

## 5. Finite Element Simulation of Embedded Sensor Performance

**Figure 10:**
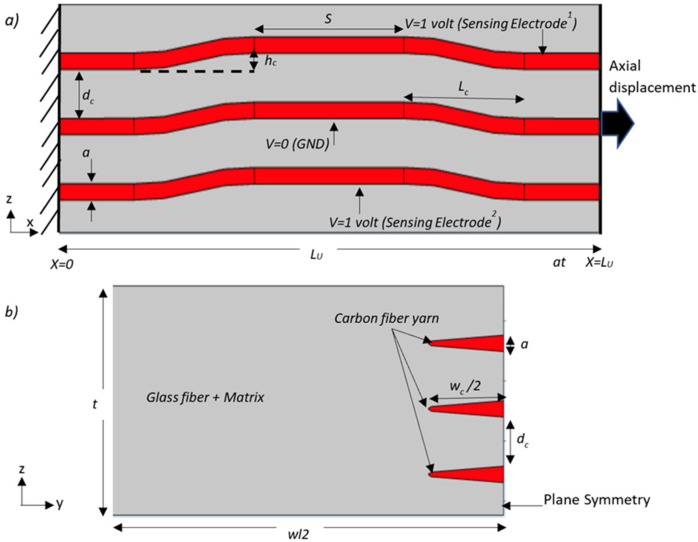
The unit cell’s main geometric parameters for integrating carbon yarn into the epoxy impregnated 2/2 twill weave glass fabric. Schematic cross-sectional diagram of (**a**) the front view and (**b**) side view of the unit cell.

To understand the behavior of the intra-ply hybrid composite, a unit cell finite element analysis (FEA) was conducted. The representative unit cell considered was extracted from the sample (B-type), as illustrated in Figure 10. To simplify the unit cell model that has a repeating element along the x-axis, the three carbon fiber yarns were assumed to be enveloped by glass fiber reinforced polymer (GFRP). The cross-sectional shape of the carbon yarn was assumed to be elliptical. The pattern of simulated yarns had the same geometry as the fabricated sample where the carbon yarn was crimped as it traveled over two and then under two glass fiber yarns. All the parameters that define this unit cell are presented in Figure 10 and detailed in Table 5. The curvilinear coordinates feature in FEA was implemented in the carbon fiber yarn to create a coordinate system following the fiber crimp angle in which the orthotropic material properties can be defined [34]. Material properties used in the model were based on values for Interglass-92125 [28,35] and carbon fiber yarn based on T300 [29], which can be seen in Table 6.

**Figure 11:**
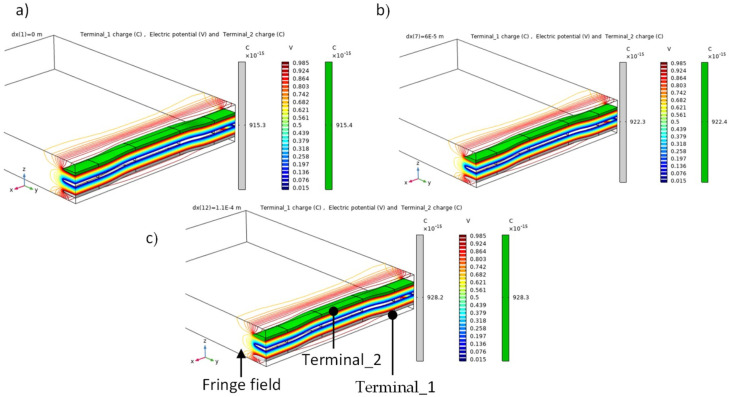
Electric potential (V) contour shows the distribution through the thickness of the unit cell at (**a**) zero axial load where the initial capacitance for terminal_1 and tramnial_2 was 0.91 pF, (**b**) 60 µm axial tensile displacement and the predicated capacitance was 0.922 pF, and (**c**) 110 µm axial tensile displacement and the predicated capacitance was 0.928 pF. To obtain capacitance (C) using FEA, the electric charge (Q) was divided by the electric potential (V = 1 V).

To provide increased confidence in the predicted results from the numerical model, the gauge factor of the capacitive sensor was calculated and compared with the experimental result. The boundary conditions that were used in the simulation for predicting the performance of the capacitive sensor are presented in Figure 10. The sensing (upper and lower) electrodes were driven by one volt, and the intermediate yarn (electrode) had an electric potential of zero volt (grounded). Using symmetry to simplify the unit cell analysis and to reduce the computing time, half the unit cell was considered, as shown in Figure 10b. The finite element analysis was carried out with COMSOL Multiphysics software. An example of the output from the finite element model is given in Figure 11, which shows how the electric potential propagated in the glass fiber reinforced polymer at zero axial loads where the initial capacitance of the unit cell was 0.91 pF. Obviously, the electric field of the capacitive sensor is restricted to the area between the electrodes and near the fringe region, which represents the non-uniform electric field around the edge of the carbon yarn. Therefore, detecting a variation in the electric field due to structural deformation or interfacial damage occurring will be highly sensitive in the area between the electrodes and the fringe region.

**Figure 12:**
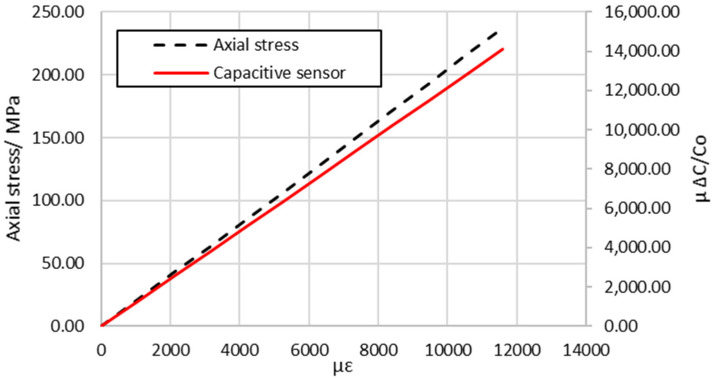
Simulated axial stress and the relative change in capacitance against the nominal strain using FEM to obtain the effective elastic modulus (Ex) and gauge factor for the capacitive sensor.

In addition to modeling the dielectric response at zero applied axial force described above, the finite element model was also used to predict the capacitive response of the unit cell as a function of axial tensile displacement. This analysis was conducted to directly compare the results of the physical characterization of the intra-ply hybrid composite structure. To simulate the axial tensile load that was introduced by the tensile test machine, forced axial displacements in the x-direction were applied at x = L_U_ of the unit cell while the opposite face was constrained (at x = 0), as shown in Figure 10a. The analysis was run over a range of applied axial tensile displacements, and the results are plotted in Figure 12, which shows that the relative change in capacitance increased linearly with the applied axial strain. Further details of the results are given in Table 7, which compares the measured gauge factor of the capacitive sensor and effective elastic modulus along the x-axis, and those predicted by the finite element model for the unit cell. It is clear that the scale of the errors was small, with the maximum difference between the observed and predicted gage factor and effective elastic modulus being only 7% and 2%, respectively.

Figure 12 illustrates that the predicted response of the capacitive sensor did not detected damage with an increase in the applied axial load compared with the experimental result as the finite element model for damage development was not considered in this analysis. The main purpose of this section is to perform a stress analysis method to evaluate and locate the damage onset that was detected by the embedded sensor for the intra-ply hybrid composite. 

The tensile test for type-B specimens in Section 4.1 shows that the turning point occurred between applied tensile strains of 1961 µɛ and 3305.7 µɛ (see Table 3). These strain values were used in the finite element model to predict the maximum stress in the carbon yarn, and the results are summarized in Table 8. The results clearly show that transverse shear stress (τ_xz_) had maximum values of 43.5 MPa and 73.5 MPa, corresponding to the axial strains of 1961 and 3305.7, respectively.

## 6. Analysis of Results and Discussion

The key novelty in the work presented is the ability to detect the damage onset for intra-ply hybrid composite material (GF/CF) using a capacitance-based sensor. To investigate this capability of the self-sensing intra-ply hybrid composite, it was necessary to conduct several experiments including uniaxial tensile and 3-point bending tests to characterize its electromechanical properties. 

Consider the experimental results presented in Figure 7 and Figure 8, which show two curves (upper and lower sensor) that initially increased with respect to the applied uniaxial stress, and then a point of inflection was reached at approximately 50 MPa and 57 MPa for the sensor configurations A and B, respectively. It is clear that the laminate stacking sequence of the intra-ply hybrid composite affected the stress value at the turning point and the mechanical properties. For example, the modulus of elasticity increased from 18.7 GPa to 20.9 GPa when ply stacking sequence of the intra-ply hybrid was changed from configuration A to B. The embedded sensor of the intra-ply hybrid composite was able to successfully measure the axial strain with a linear change in electrical capacitance. The sensor that was developed has a limited strain range up to 0.25% (turning point) but has good sensitivity of around 1.3 compared to capacitance-based sensors developed in recent years, which is in the range of 0.92 [31]. Moreover, this technique allows the structure of upper and lower plies to be monitored by two sensors, as can be seen in the result for sample B-1 where the turning point (damage onset) was first detected in the lower plies during the tensile test. This response (turning point) was also seen in the 3-point bending test for sensor configuration A, as shown in Figure 9. A series of cyclic flexural tests were performed on one sample where the applied flexural stress did not exceed 30% of flexural strength. The turning point started to appear at approximately 63.5 MPa, beyond which the relative change in capacitance decreased. Figure 9d illustrates that the specimen was loaded four times at 112.7 MPa, and the result shows the turning point that appeared when the load was applied and removed. The result indicates that the integrated capacitive sensor has good repeatability for the turning point, which is around (11,837.5 ± 185) µΔ*C*/*C_O_*.

**Figure 13:**
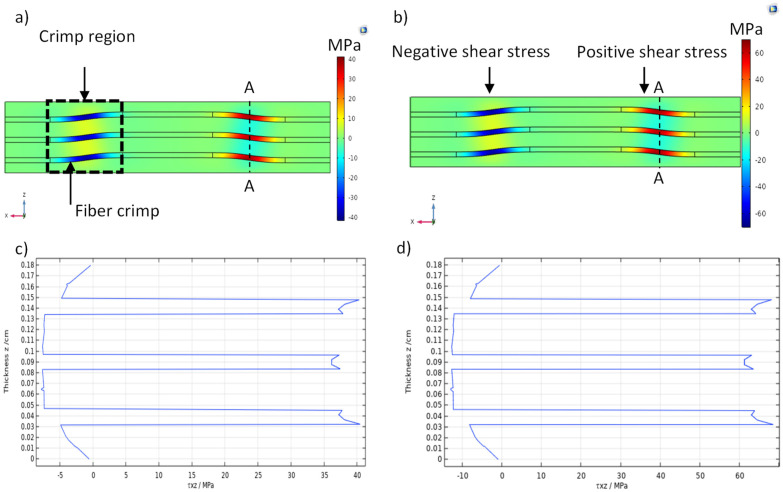
(**a**,**b**) Transverse stress contours (τ_xz_) corresponding to the applied axial tensile strains of 1961 (µɛ) and 3305.7 (µɛ), respectively. (**c**,**d**) Graph of transverse shear stress (τ_xz_) against the unit cell thickness (along section A−A) for 1961 µɛ and 3305.7 µɛ, respectively. A−A refers to transverse stress along the thickness FE model of the unit cell.

**Figure 14:**
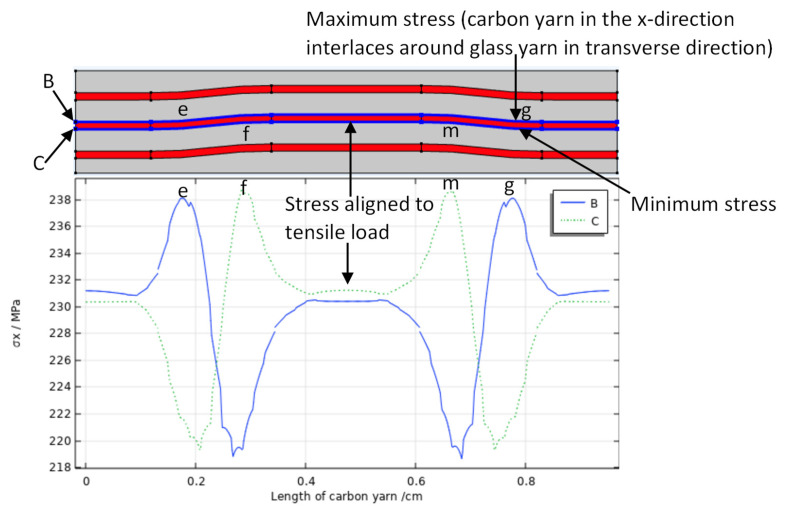
The distribution of axial stress (σ_x_) at the interface between the carbon yarn and the matrix for the applied tensile strain of 1961 µɛ. B and C refer to the axial stress along the length of carbon yarn for the upper and lower surface.

The reduction in the capacitive sensors over the turning point indicated a risk of cracking or may correlate to interfacial damage. Therefore, sample B was modeled, and critical strain values of the turning point (1961 µɛ and 3305.7 µɛ) were used in the finite element model to calculate the maximum stresses and then analyze the damage onset in the carbon fiber yarns (weft yarn). The predicated result indicated that the transverse shear stress (τ_xz_) had a critical value, as shown in Table 8. The transverse shear stress distribution of the unit cell showed that the carbon fiber yarns experienced high transverse shear stress (τ_xz_) on the middle of the crimp region, as seen in Figure 13a,b. Figure 13c,d presents the values of maximum transverse shear stress (43.5 MPa and 73.54 MPa) corresponding to 1961 µɛ and 3305.7 µɛ, respectively, which were predicted by the through-thickness FE model of the unit cell. The carbon yarn was exposed to bending in the crimp region during tensile load application, which was imposed by glass fiber reinforced polymer (warp) to the carbon yarn (weft) at points e, f, m, and g, as seen in Figure 14. This bending effect was shown in the positive and negative transverse shear stress distribution (see Figure 13b).

The most important parameter determining the delamination resistance of composites is the inter-laminar shear strength (ILSS). It has been reported that delamination can occur between the carbon fiber and matrix within a shear stress range of (40–60) MPa [37]. It is clear from the predicted result that the carbon fiber yarn is exposed to high transverse shear stress, which led to inter-fiber delamination (cracks). A potential cause of this was the crimping of the carbon fiber to form the intra-ply hybrid composite material, as shown in Figure 13a, which introduces stress concentration to the yarn. The axial stress (σ_x_) at the interface between the carbon yarn and the GFRP for an applied tensile strain of 1961 µɛ is plotted in Figure 14. It can be seen that the axial stress at the crimp region was larger than the axial stress of carbon yarns that were aligned to the applied tensile load. This high stress in the crimp region predisposes to initiate the rupture of fibers compared with the region where carbon yarn is aligned with the tensile load. However, according to the technical data sheet, the carbon fiber reinforced polymer used in this study has a tensile strength of around 1900 MPa [27]. The maximum axial stress at the carbon yarn predicted by FEA (764 MPa) is not considered to be a critical value to cause fiber fragmentation. Thus, the earlier inter-fiber (carbon fiber yarn–polymer matrix) damage, which occurred in the crimp region when the transverse shear stress became critical, led to altering the effective dielectric constant of the intra-ply hybrid composite. This effective dielectric constant at the turning point decreased, which may cause a capacitance reduction under the mechanical loading. The micro-cracks propagated with increased axial load and then several damage events occurred, with notable reduction in the capacitance until failure.

## 7. Conclusions and Outlook

A self-sensing intra-ply hybrid fiber reinforced polymer-based capacitive sensor was fabricated and characterized. The hybridization method for the intra-ply hybrid composites (carbon/glass) was described. Two types of experiment were considered to characterize the integrated capacitive sensor in the intra-ply hybrid composite to detect the onset of damage, uniaxial tensile, and three-point bending tests. Based on the experimental tests, the main findings can be presented as follows:

- The technique developed from real-time sensing has a high capability to monitor a structure’s integrity. This monitoring process can be a beneficial application for use as self-sensing for intra-ply hybrid composites.
- The smart material developed showed linear sensitivity (around 1.3), and repeatability was measured to be 185 µΔ*C*/*Co*.
- The reduction in the capacitive sensors over the turning point seen in the tensile and flexural test indicates the onset of damage.

The finite element model was used to clarify and explain the test data, and the results show:

- Measured gage factor and effective elastic modulus were in close agreement with a finite element simulation of the unit cell considered.
- The start of inter-fiber damage was in the crimp region where carbon yarn in the x-direction interlaced with glass yarn in the transverse direction.
- The turning point that was experimentally detected by an integrated capacitive sensor at around 0.25% strain may be attributed to the onset of inter-fiber cracking, which may cause critical transverse shear stress in the crimp region.

## Figures and Tables

**Table 1:** Electrical and mechanical properties. (*) Indicates properties value of the weft direction.

Property	Carbon Fiber Filament T300 [26,27]	Interglas-92125 * [28,29]
Electric resistivity/Ω·cm	1.7 × 10^−3^	---
Permittivity	13.5	4.7
Tensile Modulus/GPa	230–238	18
Tensile strength/MPa	3530–3750	320
Failure strain/%	1.5–1.6	1.8
Flexural modulus/GPa	---	24
Flexural strength/MPa	---	460

**Table 2:** Main dimensional parameters of the tensile test specimens where each specimen consisted of seven layers.

Sample Number	Thickness (*t*)/mm	Width (*w*)/mm	Length (*L*)/mm	Gauge Length (*l*)/mm
A-1	2.05	25.6	250	172
A-2	1.75	26.1	250	172
B-1	1.8	25.6	250	175
B-2	1.73	26.3	250	176

**Table 3:** Electrical and mechanical results obtained from the tensile tests for type-A and -B specimen configurations.

Sample Number		E_x/_GPa	Initial Capacitance/pF	*k* (Gauge Factor)	Turning Point Characteristics		
					$µ\frac{\Delta C}{C_{O}}$	µɛ	$\mathrm{Axial}\;\mathrm{Stress}\;(\sigma_{a})/\mathrm{MPa}$
A-1	U	18.05	52.4	0.84	2519	2411.29	46.1741
	L		58.5	0.95	2218	1960.09	40.8457
A-2	U	19.5	49.5	1.33	3436	2243.92	45.3438
	L		54.8	2.45	7848	3715.06	66.6773
Average		18.77	53.8	1.39	4005.2	2582.59	49.76
B-1	U	20.9	65.6	1.191	4160	3305.70	62.999
	L		67.6	1.098	2733	2454.58	51.4599
B-2	U	20.9	80	1.484	5710.3	2846.33	64.5453
	L		80.3	1.468	2894	1960.90	48.9629
Average		20.9	73.37	1.31	3874.5	2641.87	57

**Table 4:** Electrical and mechanical results obtained from three-point bending test for type-B specimens. R* is the ratio of the maximum flexural stress to the flexural strength of GFRP (460 MPa).

Maximum Flexural Stress/MPa	Stress Ratio/R*	Approximated Deflection (δ)/mm	Loading Frequency/mHz	Turning Point Characteristics	
				Flexural Stress/MPa	$µ\frac{\Delta C}{C_{O}}$
53.5	12	2.9	82	---	---
65.4	14.5	3.5	54	63.7	10,450
86	19	4.6	54	69.1	10,950
112.7	24.5	6	60	57.8	11,550

**Table 5:** The main parameters of the unit cell considered.

Material	Parameters
GFRP (Glass fiber + Matrix)	L_U_ = 9.5 mm, w/2 = 12.5 mm, t = 1.799 mm
Carbon yarn	a = 0.1285 mm, w_c_/2 = 0.8 mm, d_c_ = 0.3855 mm, s = 2.63 mm, h_c_ = 0.1285 mm, L_c_ = 2.12 mm

**Table 6:** Material properties of the unit cell considered.

Material	Elastic Modulus/GPa			Shear Modulus/GPa			Poisson’s Ratio			Effective Permittivity	Ref
	E_x_ (Weft)	E_y_ (Warp)	E_z_	G_xy_	G_yz_	G_xz_	*v_xy_*	*v_yz_*	*v_xz_*	$\varepsilon_{r}$	[-]
Interglass-92125	18	19	12	7.2	4	4	0.05	0.35	0.35	4.7	[28,29,35]
Carbon yarn	220	13.79	13.79	8.97	4.86	8.97	0.2	0.25	0.2	13.5	[29,36]

**Table 7:** Comparison of the measured and the finite element predicted effective elastic modulus and gauge factor.

Item	Experiment	COMSOL (FEA)	Error %
Effective elastic modulus, E_x_	20.9 GPa	20.5 GPa	2
Gauge factor, $k=\frac{\Delta C/C_{O}}{{ɛ}_{x}}$	1.31	1.217	7

**Table 8:** Finite element stress predictions for the unit cell for 1961 (µɛ) and 3305 (µɛ).

Strain (µɛ)	$\mathrm{Axial}\;\mathrm{Stress}\;(\sigma_{a})/\mathrm{MPa}$	Maximum Stress at Carbon Yarn/MPa					
		$\mathrm{Axial}-\mathrm{Stress}\;\sigma_{x}$	Transverse Normal Stress		Transverse Shear Stress		
			$\sigma_{y}$	$\sigma_{z}$	τ_xz_	τ_xy_	τ_yz_
1961	39.6	453	6.5	6.0	43.5	2.3	2.5
3305.7	66.9	764	11	10.1	73.5	4.0	4.2

## Data Availability

All the available data are presented within the manuscript.
